# Comment on “Dying in the Sun: Direct evidence for elevated UV-B radiation at the end-Permian mass extinction”

**DOI:** 10.1126/sciadv.adi0570

**Published:** 2023-08-25

**Authors:** Alistair W. R. Seddon, Boris Zimmermann

**Affiliations:** ^1^ Department of Biological Sciences and Bjerknes Center for Climate Research, University of Bergen, Bergen, Norway.; ^2^ Norwegian University of Life Sciences, Ås, Norway.

## Abstract

Liu *et al.* present evidence of increased abundance of UV-B–absorbing compounds in fossilized sporomorphs at the end-Permian mass extinction based on Fourier transform infrared (FTIR) spectroscopy. Their approach assumes that UV-B–absorbing compounds are present in the fossilized sporomorphs spanning the extinction interval and that they can be quantified using FTIR. However, further analysis reveals that the signal that they aim to focus on is weak and poorly resolved against nonrandom background interference most likely associated with water vapor. We also show that the peak detection methods that they use are inappropriate for use on these fossil sporomorphs because their methods select only 3.9% of the spectra at the target waveband of interest. The reconstruction that they present is based on baseline variations in the spectra and cannot be confidently attributed to variations in UV-B–absorbing compounds. “Direct” evidence for UV-B radiation at the end-Permian mass extinction cannot be claimed to have been observed in this record.

## Teaser

Methods used by Liu* et al.*&nbsp;were inappropriate to reconstruct UV-B radiation during the end-Permian mass extinction.

**Fig. 1.**
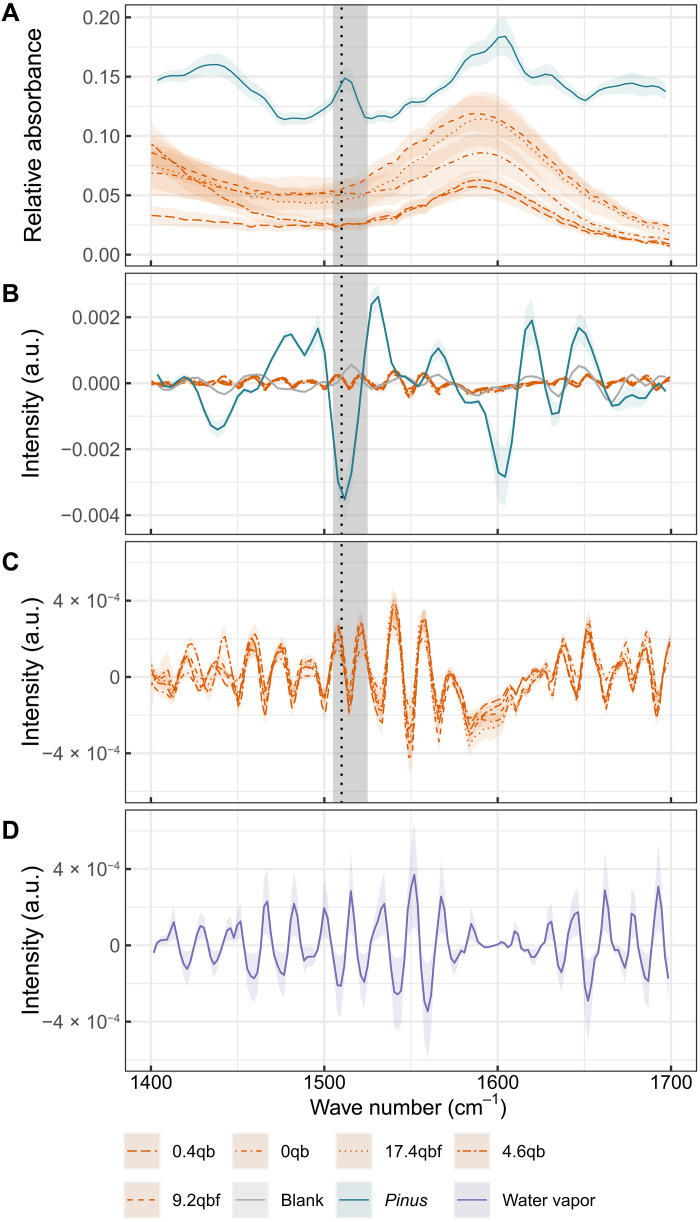
FTIR spectra from sporomorphs and water vapor. (**A**) Baseline-corrected mean spectra for the region of interest for five sediment samples taken from the work of Liu *et al.* (*2*) (samples 0.4qb to 9.2qbf). The dashed line shows the target band to extract a peak at 1510 cm^−1^, and the shaded area represents the window used to search for the maximum absorbance value by Liu *et al.* (see table 1 in the work of Liu *et al.* for sample size underlying each mean spectrum). A typical mean FTIR spectrum from three *Pinus* spp. pollen grains taken from a Holocene sediment core [data from (*4*)] is also presented using the same baseline correction as Liu *et al.* The relative absorbance of the mean spectrum from *Pinus* pollen grains has been shifted by an arbitrary value (0.1) to allow for comparison. (**B**) Typical second derivative FTIR spectrum from three fossil *Pinus* spp. pollen grains [data from (*4*)]. The dashed line shows the target waveband to extract a peak at 1510 cm^−1^, and the shaded area represents the window used to search for the maximum absorbance value by Liu *et al.* Mean second derivative spectra of the five samples in (A) and the atmospheric blank from (*4*) are also plotted for comparison. (**C**) Mean second derivative FTIR spectra for sporomorphs from the five sediment samples in (A). (**D**) Mean second derivative of three pure water vapor spectra from (*9*). All second derivative spectra are calculated using a Savitzky-Golay filter (second degree polynomial and window size of 9). Shading surrounding the spectra represents the 95% confidence intervals. a.u., arbitrary units.

Exposure to ultraviolet-B (UV-B) radiation can induce a set of chemical responses in plants, including the production of UV-B–absorbing compounds in the sporopollenin of pollen and spores (*1*). Liu *et al.* (*2*) present evidence of increased abundance of UV-B–absorbing compounds in fossilized sporomorphs at the end-Permian mass extinction using Fourier transform infrared (FTIR) spectroscopy. Their analysis targets the variations in the absorbance band at 1510 cm^−1^. This band is present in FTIR spectra from both modern and subfossil pollen (Fig. 1A) (*3*, *4*) and, in cases of exceptional preservation, fossilized sporomorphs (*5*). This absorbance band is the result of the stretching of aromatic (phenyl ring) bonds, which are characteristic of UV-B–absorbing compounds such as *para*-coumaric acid and ferulic acid (*6*, *7*).

If such a peak is to be extracted for quantitative FTIR spectroscopy and then also confidently ascribed to the stretching of aromatic ring bonds, then it should be observable in the region close to 1510 cm^−1^ and show minimal interference with other compounds (*8*). However, peaks at 1510 cm^−1^ that are characteristic of other subfossil and modern pollen FTIR spectra, which would be appropriate for use in quantitative infrared spectroscopy [e.g., Fig. 1 (A and B)], are not present in these fossil samples. The peak at 1510 cm^−1^ is not present in the second derivative spectra from most the samples (Fig. 1C). A peak at 1515 cm^−1^ does exist, but the magnitude of this peak is generally not larger than surrounding interference signals. Instead, the second derivative spectra from Liu *et al.* show the diagnostic features of water vapor (note the rotational-vibrational H-O-H scissoring band centered around 1620 cm^−1^; Fig. 1, C and D) (*9*–*11*). Any peaks in the region of interest cannot be differentiated from nonrandom noise.

If the effect of water vapor was consistent across samples, then post hoc correction procedures [e.g. (*11*)] can be used to remove interference before peak detection. However, such methods require measurements of water vapor at the time of sample collection. Similarly, if the interference signals are weak compared to the strong absorbance of the samples, then water vapor interference may have a minimal impact on peak detection. In the case of Liu *et al.*, the target peak at 1510 cm^−1^ is not distinguishable from the water vapor–influenced nonrandom noise. The fact that significant correlations exist between second derivative peaks between 1750 and 1700 cm^−1^ (related to water vapor interference) and between the proposed aromatic peak (between 1525 and 1505 cm^−1^) confirms that such interference cannot be discounted (e.g., the Pearson’s *r* correlation coefficient between the aromatic peak and the peak at 1725 cm^−1^ using the samples presented in Fig. 1C is equal to 0.44; *P* < 0.000001; *N* = 131).

**Fig. 2.**
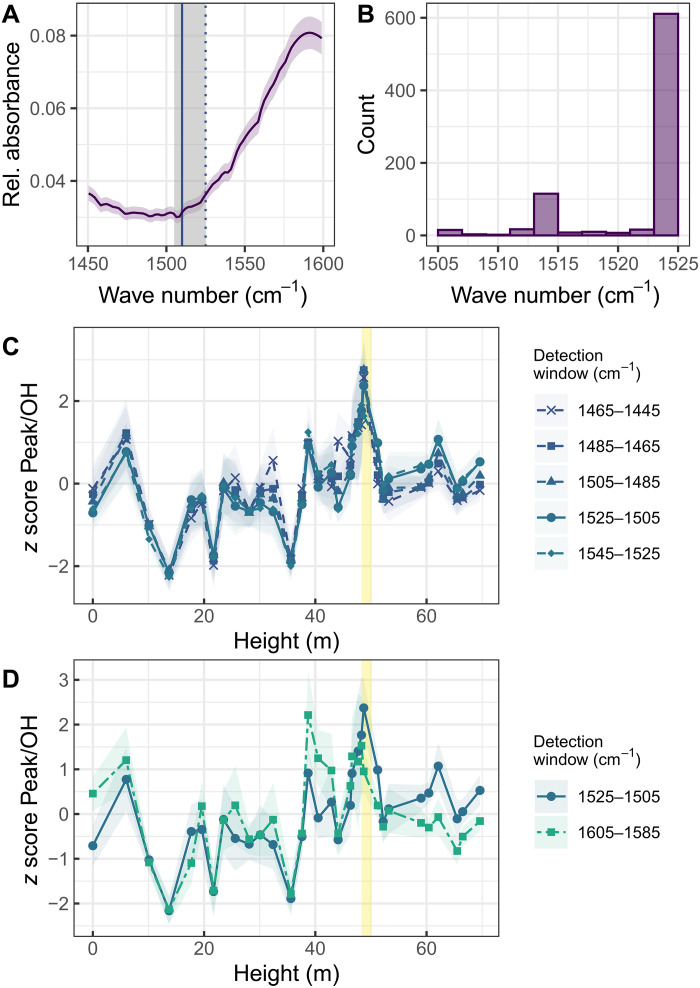
Artifacts in the peak detection algorithm. (**A**) Mean of all spectra for the 1600- to 1450-cm^−1^ wave numbers. The purple shading reflects the 95% confidence interval. The gray shaded area is the window used by Liu *et al.* (*2*) for peak selection, and the filled gray line shows the target band to extract a peak at 1510 cm^−1^. The dashed line is the point at the end of the window, intersecting the shoulder of the broader peak around 1590 cm^−1^. (**B**) Histogram to show which wave number is selected as representing the maximum absorbance following the peak selection method used in the paper using the vetted data. The majority of grains have a wave number selected at the upper limit of the window, not at the proposed 1510-cm^−1^ peak. (**C**) Standardized *z* score means of the peak absorbance/OH peak calculated for different wave number windows spanning 20 cm^−1^. The strongly overlapping curves indicate that the reconstructions are a feature of the baseline variations of the spectra rather than a specific peak related to aromatic UV-absorbing compounds. The yellow band represents the marine extinction interval at the end-Permian mass extinction, as highlighted in the work of Liu *et al.* Colored shading reflects the 95% confidence intervals. (**D**) Comparison of reconstructions (using *z* score means) of the “narrow aromatic” and “broad aromatic” wavebands using peak detection windows 1525 to 1505 cm^−1^ and 1605 to 1585 cm^−1^. The yellow band represents the marine extinction interval at the end-Permian mass extinction, as highlighted in the work of Liu *et al.* Colored shading reflects the 95% confidence intervals.

The low signal-to-noise ratio compared to water vapor interference means that the peak detection method used by Liu *et al.* is inappropriate. Their approach searches for the maximum absorbance within a window between 1525 and 1505 cm^−1^ (Fig. 2A). Such a method is conditional that the waveband at 1510 cm^−1^ is narrow and distinguishable from noise. However, although they state that a weak peak related to UV-B–absorbing compounds is “centred at 1510 cm^-1^,” using their method, only 3.9% of all the spectra have maximum absorbance at this target waveband (2.7% when only the “vetted” spectra are used). For 69.9% of the spectra presented in their paper (60.1% of the vetted spectra), maximum absorbance occurs at the upper limits of the window, associated with the shoulder of a broader peak at 1590 cm^−1^ (Fig. 2B). Because we can recreate the same or very similar stratigraphic pattern using different 20-cm^−1^ windows between 1545 and 1445 cm^−1^, the reconstruction cannot be confidently attributed to variations in UV-B–absorbing compounds (Fig. 2C). Their reconstructions are based on general variations in the baseline of the spectra, rather than a peak that can be confidently attributed to the stretching of aromatic ring bonds.

Liu *et al.* acknowledge the weak absorbance bands at 1510 cm^−1^ and highlight the chemical changes linked to diagenetic processes. Diagenesis likely results in the repolymerization of sporopollenin into a geopolymer, which is chemically distinct from modern and subfossilized sporomorphs (*12*). Infrared spectra from fossil sporomorphs therefore tend to be dominated by a broad aromatic band at 1590 cm^−1^ (*13*). They suggest that a correlation between the absorbance at the “narrow” 1510-cm^−1^ waveband and this broader waveband confirms that both regions are representative of analytes with UV-B–absorbing properties.

However, a strong correlation between the “broad” (i.e., 1590 cm^−1^) and narrow (1510 cm^−1^) wavebands is to be expected using their methods. Both bands are sampled from the same peak and so cannot be considered independent. Furthermore, although the peak at 1590 cm^−1^ can be described as an aromatic peak, it is nonspecific. Repolymerization may result in UV-B–absorbing compounds being represented by this broader peak in fossil spectra, but this peak is also likely to contain diagenetic products from other organic constituents of pollen (including plant waxes and lipids), as well as products linked to thermal maturation (*14*, *15*). If the sensitivity tests presented by Liu *et al.* are robust and the two wavebands are equivalent, then there should also be a transient response in the relative absorbance of this broad waveband at the end-Permian mass extinction. However, the transient response at the extinction window is not distinguishable from a large excursion at a height of ~40 m when the broad waveband between 1605 and 1585 cm^−1^ is used (Fig. 2D).

Our analyses cast doubt on the claims made in the original paper. Liu *et al.* state that peaks in the spectra are centered at 1510 cm^−1^, but this is not true for the majority of cases. Any peaks found in the region of the 1510-cm^−1^ waveband are weak and poorly resolved against background interference related to water vapor, such that the peak quantification method is inappropriate. Because it is not possible to attribute reconstructions specifically to the stretching of aromatic ring bonds related to UV-B compounds, “direct” evidence of increased UV-B radiation at the end-Permian mass extinction has not been observed in this record. We caution against others using similar methods to process infrared fossil pollen spectra unless the main criteria for quantitative spectroscopy are fulfilled.
